# Colloidal synthesis and etching yield monodisperse plasmonic quasi-spherical Mg nanoparticles†

**DOI:** 10.1039/d5nh00205b

**Published:** 2025-05-22

**Authors:** Andrey Ten, Christina Boukouvala, Vladimir Lomonosov, Emilie Ringe

**Affiliations:** a Department of Materials Science and Metallurgy, University of Cambridge 27 Charles Babbage Road Cambridge CB3 0FS UK vl318@cam.ac.uk er407@cam.ac.uk; b Department of Earth Sciences, University of Cambridge Downing Street Cambridge CB2 3EQ UK

## Abstract

Mg is a low-cost, earth-abundant, and biocompatible plasmonic metal. Fine tuning of its optical response, required for successful light-harvesting applications, can be achieved by controlling Mg nanoparticle size and shape. Mg's hexagonal close packed crystal structure leads to the formation of a variety of unique shapes in colloidal synthesis, ranging from single crystalline hexagonal platelets to twinned rods. Yet, shape control in colloidal Mg nanoparticle synthesis is challenging due to complex nucleation and growth kinetics. Here, we present an approach to manipulate Mg nanoparticle shape by one-pot synthesis followed by colloidal etching with polycyclic aromatic hydrocarbons. We demonstrate how tips and edges in faceted Mg nanoparticles can be preferentially etched to produce quasi-spherical nanoparticles with smooth surfaces. The developed approach provides an essential shape control tool in colloidal Mg synthesis potentially applicable to other oxidising metals.

## Abstract 2

Etching of faceted nanoparticles with polycyclic aromatic hydrocarbons provides an oxygen-free route to monodisperse colloids, as exemplified with plasmonic Mg structures.

## Graphical abstract

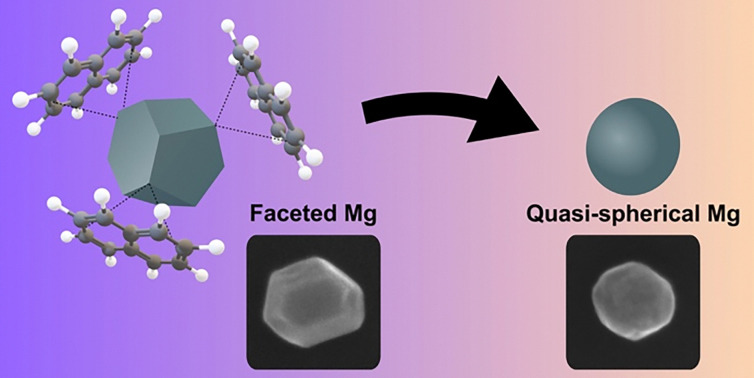

**New concepts**

The size and shape of nano-objects determine their properties, including localised surface plasmon resonance energy in plasmonic metals. Such dependence provides a large tuning range but exerts strong pressures on the homogeneity of materials. This paper reports an approach for colloidal etching of metallic nanoparticles that leads to monodisperse quasi-spherical structures. Specifically, the etching is performed by complexation of the surface metal atoms with polycyclic aromatic hydrocarbons, an approach departing from the established oxygen-based etching. This new concept enables the manipulation of shape in oxidation-prone metals, which we exemplify here by transforming faceted nanostructures of Mg into smooth quasi-spheres. We then show that the metallic character and associated plasmonic response of Mg nanoparticles are maintained through the etching process. We anticipate that this concept of oxygen-free etching will unlock the synthesis of new shapes in a monodisperse fashion for other oxidation-sensitive metallic nanoparticles.

Mg nanostructures sustain localised surface plasmon resonances (LSPRs), coherent oscillations of free electrons driven by an electric field. LSPRs have been traditionally studied and utilised in Au and Ag nanostructures but more recently alternative metals such as Cu,^1,2^ Al,^3,4^ and Mg^5^ have been investigated.^6^ Mg stands out for its inherent biocompatibility, low cost, earth-abundance, and competitive plasmonic performance spanning ultraviolet, visible, and near-infrared (UV-vis-NIR) frequencies.^5^ Consequently, a wide range of applications including optical devices,^7,8^ sensing,^9–13^ light-enhanced catalysis,^14–16^ surface-enhanced Raman scattering,^16^ and photothermal heating^17,18^ are explored to utilise Mg's plasmonic properties.

Controllable and cost-effective synthetic approaches are essential to leverage Mg's advantages over other plasmonic metals. Colloidal synthesis is a scalable bottom-up approach that produces nanoparticles (NPs) that can be easily stored, modified, and transported.^19^ In colloidal synthesis, the size and shape of NPs are affected by several factors including the type of precursor, reducing agent, and capping ligand, as well as reaction conditions.^20,21^ In addition, synthesis can be performed in multiple steps, for instance *via* seed-mediated growth to further enable tuning of size and shape.^22–24^

The reduction of an Mg halide with an alkali metal has first been demonstrated in the 1970s by Rieke *et al.*,^25,26^ and inspired recent explorations for the colloidal synthesis of Mg NPs by reducing di-*n*-butyl magnesium (MgBu_2_) with lithium in the presence of a polycyclic aromatic hydrocarbon electron carrier.^27–31^ The synthetic details are now established for Mg single crystalline hexagonal platelets and twinned rods of 80 to 1300 nm.^28^ A seed-mediated growth method for Mg NPs was also reported recently, yielding faceted Mg NPs with narrow size distribution.^29^ Colloidally synthesised Mg NPs remain stable and plasmonic in air up to 400 °C owing to the spontaneous formation of a protective, self-limiting surface oxide layer.^32^

The plasmonic properties of NPs are directly affected by their shape and size. NPs adopt a shape that either minimises their thermodynamic surface energy (thermodynamic regime) or is controlled by facet-specific growth rate (kinetic regime) as captured by various Wulff constructions.^33,34^ Colloidally synthesised Mg NPs naturally form faceted shapes, as do many metallic NPs.^35,36^ Of the various shapes, spheres provide the most homogeneous optical response, which is particularly important for applications involving NP self-assemblies or aggregates.^37,38^ Oxidative etching is a common approach to produce metallic nanospheres.^39,40^ Particularly, halide ions in the presence of oxygen can selectively etch low-coordinated surface atoms of Au,^41–47^ Ag,^48,49^ Pd,^50^ Pt,^51^ and Rh^52^ NPs, leading to corner rounding and aspect ratio reduction. Roundness can be further improved by iterative growth and etching steps.^45–47^ Under certain conditions, oxidative etching can even selectively remove twinned NPs^53–55^ and slow down growth kinetics,^56^ making it an effective method to control colloidal synthesis.^40^ Balancing oxidative processes with reductive steps allows for the formation of NPs with unique shapes and sizes.^41–59^ However, oxidative etching cannot be used for readily oxidising metals due the formation of a surface oxide layer that prevents further morphological modifications.

Here, we report a one-pot colloidal etching approach akin to oxidative etching using polycyclic aromatic hydrocarbons in the absence of oxygen. When applied to Mg colloidal synthesis, this methodology transforms faceted Mg NPs into monodisperse quasi-spherical Mg NPs. We first investigate the reaction dynamics of seed-mediated growth of Mg to establish when the reduction slows down sufficiently to enable a successive etching step. We then find that the addition of excess naphthalene to colloidal Mg NP synthesis selectively etches edges and corners of faceted Mg NPs and produces plasmonic quasi-spherical Mg NPs. The etching mechanism reported here is a promising method for shape-controlled synthesis of oxidising metals such as Mg, and provides an approach for reproducible and monodisperse NPs.

The seed-mediated growth of faceted NPs and subsequent etching (leading to rounding) into quasi-spheres were conducted under an inert atmosphere in one pot. The reaction scheme (Fig. 1) consists of two reduction stages leading to monodisperse faceted Mg NPs, followed by the etching stage where the NPs are transformed into smooth quasi-spheres. Poly(vinyl pyrrolidone) (PVP) was added from the beginning of the synthesis to minimise NP aggregation. The effects of PVP in Mg NP synthesis have been studied by Wayman *et al.*^30^ The reaction was quenched with acetone at different times to analyse the reaction progress. The resulting NPs were imaged with scanning electron microscopy (SEM) and their average size was measured by fitting over a hundred NPs per sample to an ellipse using an automated procedure and obtaining their major axis lengths (details in ESI†). The aspect ratios in all cases were close to 1 and in the following text we will describe this length as diameter.

**Fig. 1:**
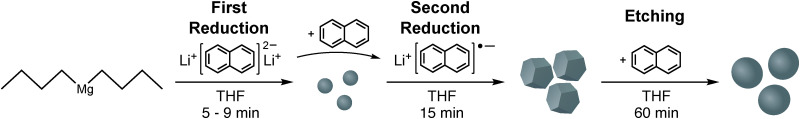
Reaction schematic for the colloidal synthesis of quasi-spherical Mg NPs.

The first stage in the growth of faceted Mg NPs involves reducing MgBu_2_ with lithium naphthalene dianion (Li_2_Napht). The highly negative reduction potential of Li_2_Napht, comparable to that of Li^0^ (reduction potential of Li is *E*^0^ = −3.04 V),^60^ generated monodisperse Mg NPs. The nucleation and growth dynamics in this step were tracked by collecting aliquots from the same flask over the course of the reaction (Fig. 2). After 3 min of reaction, NPs were grown to 57 ± 9 nm (diameter ± standard deviation) and continued growing to 84 ± 6 nm after 11 min (Fig. 2A, D, and Fig. S1, ESI†). At shorter reaction times, SEM and transmission electron microscopy (TEM) images suggest the presence of smaller NPs. However, their size could not be measured reliably because Mg NPs were aggregated and covered by surrounding contaminants produced from reaction quenching when significant MgBu_2_ is present. The low atomic number (*Z*) of Mg produces limited contrast between the NPs and the contaminants. Additionally, surface oxidation in NPs <50 nm leads to a majority oxide volume and could be associated with shape and size changes, leading to unrepresentative images. Between 3 and 11 min, both the reaction yield (conversion of the organometallic Mg^2+^ precursor into solid Mg) obtained from inductively coupled plasma optical emission spectroscopy (ICP-OES) and the average volume (ESI†) of a single NP increased linearly (Fig. 2E and Table S1, ESI†). Together, these trends confirm that Mg ions reduced to metal during this period are mainly involved in the growth of NPs, *i.e.*, that nucleation wanes by 3 min. Longer reactions in the first reduction stage yielded a bimodal colloid, as large hexagonal platelets begin forming in the reaction mixture beyond 11 min (Fig. S2, ESI†), an outcome previously reported when Li_2_Napht depletes to ∼30% of its initial concentration.^29^

**Fig. 2:**
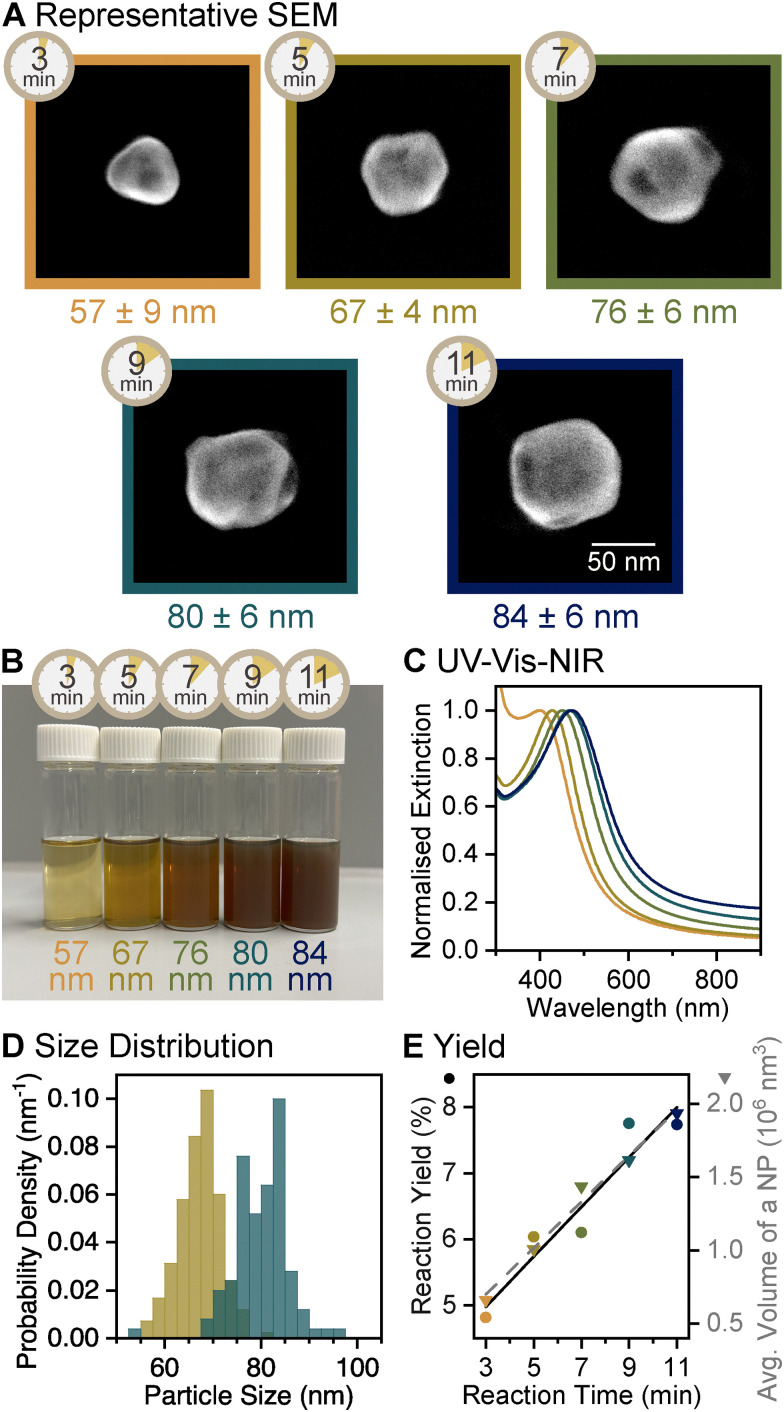
Reaction evolution of the first reduction stage of the growth of faceted Mg NPs, sampled between 3 and 11 min of reaction time. (A) SEM images of representative NPs, labelled with the reaction time as well as average diameter and its standard deviation. (B) Colloidal Mg NPs and (C) their UV-vis-NIR extinction spectra. (D) Size distribution histogram showing the diameter of Mg NPs obtained from SEM images after 5 and 9 min of reaction, *N* = 166 and 100, respectively. Equivalent data for other reaction times are reported in Fig. S1 (ESI†). (E) Reaction yield from ICP-OES (circles, black solid line) and average NP volume from SEM images (triangles, grey dashed line) as a function of reaction time showing NP growth; lines represent the linear best fit.

The Mg NPs obtained after one reduction step were plasmonic. The presence of Mg^0^ and long-term air stability were confirmed by powder X-ray diffraction (XRD) of dried Mg NPs in ambient air, revealing peaks corresponding to hexagonal close packed (HCP) Mg (Fig. S3, ESI†). The UV-vis-NIR extinction spectrum displayed an LSPR peak that redshifted from 400 to 473 nm and became more intense as NPs grew from 57 to 84 nm in diameter (Fig. 2C). The narrowness of this peak indicates homogeneity in size and shape as well as high dispersity of the NPs (*i.e.*, low aggregation). With resonances in the visible region, the colloids displayed strong colours ranging from yellow to brownish red with increasing NP size (Fig. 2B); however, this distinct colour change is in large parts due to an increase in extinction caused by larger and more concentrated NPs (Fig. S4, ESI†), with a small contribution from the 73 nm LSPR redshift.

Converting Li_2_Napht to lithium naphthalene radical anion (LiNapht, *E*^0^ = −2.73 V, from Wawzonek and Laitinen^61^ and Meerholz and Heinze^62^) leads to uniform growth of the Mg NPs formed at the first reduction stage and suppresses the formation of a bimodal colloid.^29^ The addition of stoichiometric quantities of naphthalene instantly converted Li_2_Napht into LiNapht, as confirmed by the change in colour from purple to dark green.^29,63^ The timing of naphthalene injection, *i.e.*, the start of the second reduction stage, influences the outcome obtained after 1 h of growth. Injection of naphthalene within the first 5 min of the first stage produced a mixture of faceted NPs with single crystalline and twinned Mg platelets (Fig. S5, ESI†). The presence of the latter indicates the formation of new nuclei in LiNapht environment. Indeed, starting the reaction directly from the second stage (only LiNapht, no Li_2_Napht) also produces such Mg platelets.^28^ Therefore, the precursor concentration must be sufficiently lowered before the Li_2_Napht to LiNapht conversion to avoid nucleation at the second stage. Meanwhile, conversion between 5 and 9 min of the first stage produced monodisperse Mg NPs (Fig. S6, ESI†). Lastly, conversion beyond 9 min resulted in bimodal colloids (Fig. S5, ESI†), grown from the mixture of faceted NPs and hexagonal platelets produced from longer reaction times (over 9 min) in the first stage (Fig. S2, ESI†).

The growth dynamics over the course of the second reduction stage, following a 5 min reaction in the first stage, revealed that Mg NPs grow steadily for 15 min, then the reduction considerably slows down. The diameter of NPs doubled from 82 ± 8 (1 min) to 170 ± 17 nm within the first 15 min and remained constant thereafter as measured from SEM images and confirmed by DLS (Fig. 3, Fig. S7, S8, and Table S2, ESI†). Similar trends were observed in reaction yield and average single NP volume: both increased rapidly in the first 10 min and plateaued by 20 min (Fig. 3E). The overlap of these trends confirms previous observations that reduced Mg atoms contribute to the growth of existing Mg NPs rather than nucleating new NPs. By 10 min of the second stage, the yield reached 28%, much higher than the 1% yield obtained in a single-step reduction of MgBu_2_ with LiNapht, reported previously for the same reaction time.^30^ The rate of reduction in the seed-mediated synthesis is thus much higher compared to that of the single-step approach.

**Fig. 3:**
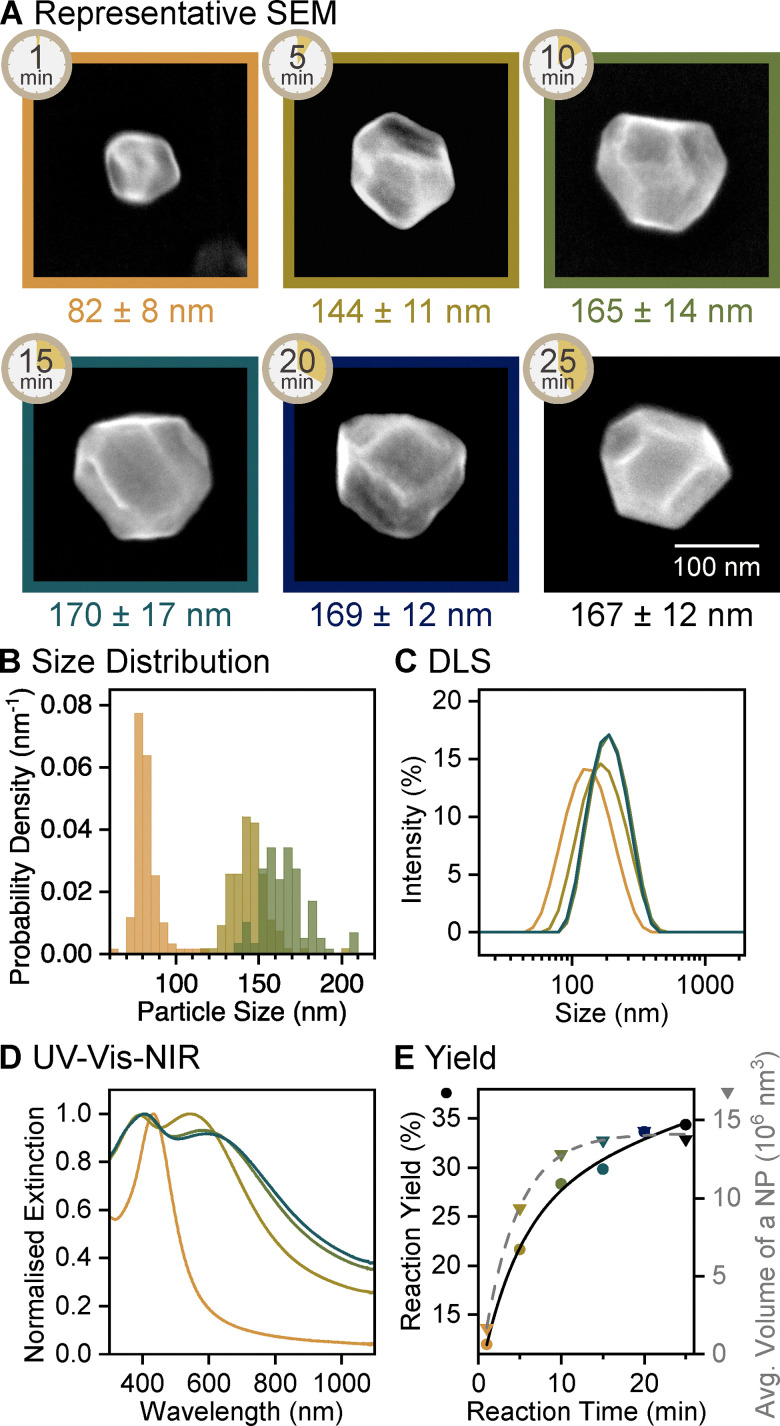
Reaction evolution of the second reduction stage of the growth of faceted Mg NPs, sampled between 1 and 25 min of reaction time within this stage, following a 5 min reaction in the first reduction stage. (A) SEM images of representative NPs, labelled with the reaction time as well as average diameter and its standard deviation. (B) Size distribution histogram showing the diameter of Mg NPs obtained from SEM images after 1, 5, and 10 min of reaction, *N* = 119, 109, and 117, respectively. Equivalent data for other reaction times are reported in Fig. S7 (ESI†). (C) Size distribution of Mg NPs after 1, 5, 10, and 15 min of reaction obtained from DLS and (D) their UV-vis-NIR extinction spectra. Equivalent data for other reactions are reported in Fig. S8 and S9 (ESI†). (E) Reaction yield from ICP-OES (circles, black solid line) and average NP volume measured from SEM images (triangles, grey dashed line) as a function of reaction time; lines represent the best exponential (association) fit.

Mg NPs remain Mg^0^ throughout their growth. The dipolar LSPR mode in their UV-vis-NIR extinction spectra (Fig. 3D and Fig. S9, ESI†) redshifted to 600 nm as NPs grew to their final size. A quadrupolar mode, characterised by an extinction maximum around 400 nm, appeared as NPs reached 144 ± 11 nm in diameter, after 10 min of growth. Such changes to the extinction profiles indicate the growth of Mg metal (Fig. S10, ESI†); an increase of the oxide layer thickness resulting in the same size change was ruled out as we calculated it would cause a <100 nm redshift and no dominant quadrupolar mode (Fig. S11, ESI†). The presence of Mg^0^ and stability of the final Mg NPs were further confirmed by obtaining clear HCP Mg XRD pattern from a freshly dried sample and the same sample left in ambient air for 15 days (Fig. S3, ESI†).

Naphthalene and anthracene can etch Mg NPs. Either of the two polycyclic aromatic hydrocarbons was added to the reaction mixture in equal amounts as the reducing agent after 60 min of second stage reaction, when no Mg yield change was detected. Naphthalene addition caused etching of the sharp edges and corners in faceted NPs. Under stirring with naphthalene, faceted NPs of 166 ± 12 nm were etched into quasi-spheres of 154 ± 14 nm in diameter over the course of 60 min at room temperature, as measured by SEM (Fig. 4A, B and Fig. S12, ESI†). The resulting Mg NPs had no discernible extended flat facets, edges, or corners (Fig. 4C and Fig. S13, ESI†). Equivalent experiments with anthracene as the etchant resulted in significant dissolution of Mg NPs. After 60 min, the amount of Mg considerably decreased and NP surface was rough (Fig. S14, ESI†), indicating a rapid and non-uniform etching that is more aggressive than with naphthalene. The extent of etching with anthracene further increased at higher reaction temperature (Fig. S14, ESI†). By contrast, for naphthalene, increasing the temperature to 40 °C led to further growth of Mg NPs instead of etching, indicating that the rate of etching with naphthalene is outpaced by the promoted growth rate.

**Fig. 4:**
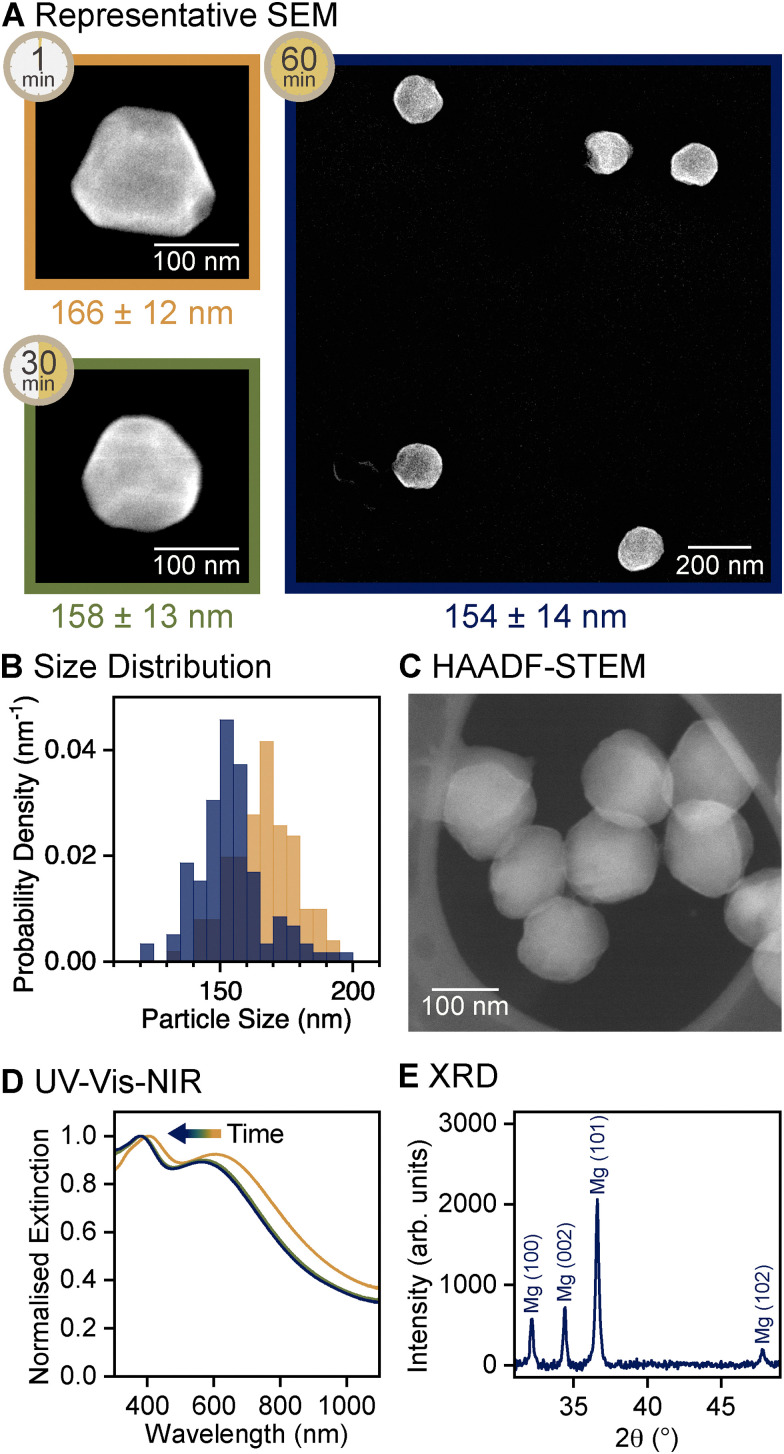
Reaction evolution and outcome of the etching stage in quasi-spherical Mg NP synthesis, sampled between 1 and 60 min of reaction time within this stage, following 5 and 60 min reaction times in first and second reduction stages, respectively. (A) SEM images of representative NPs, labelled with the reaction time as well as average diameter and its standard deviation. (B) Size distribution showing the diameter of Mg NPs obtained from SEM images after 1 and 60 min of reaction, *N* = 101 and 119, respectively. Equivalent data after 30 min of reaction are reported in Fig. S12 (ESI†). (C) High-angle annular dark-field scanning TEM (HAADF-STEM) image of quasi-spherical Mg NPs. (D) UV-vis-NIR extinction spectra after 1, 30, and 60 min of reaction. (E) XRD pattern of quasi-spherical Mg NPs after 60 min of reaction, displaying peaks corresponding to HCP Mg (PDF 00-035-0821).

The etching process likely occurs by redissolution of Mg *via* complex formation with naphthalene or anthracene. Complexing of Mg with polycyclic aromatic hydrocarbons has been reported with anthracene in THF,^64^ and is expected to occur for naphthalene based on their chemical similarity. During etching with naphthalene, the volume of Mg NPs decreased by 15% (Table 1). This volume loss was consistent with the 14% decrease, from 0.81 to 0.70 mg mL^−1^, in amount of Mg in the colloid measured by ICP-OES. Therefore, etched Mg is understood to have been redissolved in the solution and subsequently removed during centrifugation and resuspension steps.

**Table 1:** Average NP size, volume, and number concentration within the colloid throughout the etching stage of quasi-spherical Mg NP synthesis, determined by SEM and ICP-OES

Reaction time in rounding stage (min)	Average NP size (diameter, nm)	Average NP volume (nm^3^)	Concentration of NPs (NPs per mL)
1	166 ± 12	1.36 × 10^7^	3.44 × 10^10^
30	158 ± 13	1.20 × 10^7^	3.49 × 10^10^
60	154 ± 14	1.15 × 10^7^	3.53 × 10^10^

Colloidal etching with naphthalene and anthracene occurs only for Mg^0^, as would be expected from the formation of a complex. Indeed, post-synthetic addition of naphthalene and anthracene after oxide formation (through quenching and cleaning) did not result in shape change (Fig. S15, ESI†).

Mg NPs consisted mainly of Mg^0^ after etching with naphthalene, as confirmed by XRD (Fig. 4E), and therefore remained plasmonic (Fig. 4D). The decrease in the NP size upon etching is reflected by the blueshift of the dipolar LSPR peak in the UV-vis-NIR extinction spectra (Fig. 4D), as shape change alone does not lead to such shift (Fig. S16, ESI†).

In this work, we described a one-pot synthetic approach for colloidal etching of Mg NPs by redissolution of Mg^0^ in the presence of polycyclic aromatic hydrocarbons in the absence of oxygen. Naphthalene was incorporated in the third stage of the reaction to preferentially etch corners and edges of faceted NPs to yield smooth quasi-spherical Mg NPs. The approach presented here provides an important shape control tool in colloidal NP synthesis. The resulting protocol produces quasi-spherical Mg NPs, a notable addition to the Mg NP library, as the most isotropic colloidal Mg NP available to date.

## Author contributions

Andrey Ten: data curation, formal analysis, investigation, methodology, software, validation, writing – original draft, writing – review & editing. Christina Boukouvala: formal analysis, methodology, software, writing – original draft. Vladimir Lomonosov: conceptualization, investigation, methodology, project administration, supervision, validation, writing – original draft, writing – review & editing. Emilie Ringe: funding acquisition, investigation, project administration, resources, supervision, writing – original draft, writing – review & editing.

## Conflicts of interest

There are no conflicts of interest to declare.

## Supplementary Material

NH-010-D5NH00205B-s001

## Data Availability

Additional experimental and analytical details, calculated average volumes and numbers, SEM images, size distributions from SEM and DLS, photographs, UV-vis-NIR spectra, XRD patterns, shape used for numerical calculations, and simulated extinction cross sections of Mg NPs supporting this article have been included as part of the ESI.† All SEM images used for size measurements are available in Cambridge University's Apollo repository at https://doi.org/10.17863/CAM.117186. The code for Stratify^65^ can be found at https://gitlab.com/iliarasskazov/stratify. The version of the code employed for this study is version 1.1. The code for DDSCAT^66^ can be found at https://ddscat.wikidot.com. The version of the code employed for this study is version 7.3.2.
