# Supplementary material for: Colloidal synthesis and etching yield monodisperse plasmonic quasi-spherical Mg nanoparticles
Source: Nanoscale Horiz. 2025 May 22;10(8):1724–30. doi: 10.1039/d5nh00205b (PMC12147706; doi:10.1039/d5nh00205b)
Supplement: NH-010-D5NH00205B-s001 [file NH-010-D5NH00205B-s001.pdf]

Supplementary Information (SI) for Nanoscale Horizons.
This journal is © The Royal Society of Chemistry 2025

Electronic supplementary information (ESI) for Colloidal synthesis and etching yield monodisperse plasmonic quasi-spherical Mg nanoparticles

Andrey Ten,[a,b] *Christina Boukouvala,*[a,b] *Vladimir Lomonosov,*[a,b]* *and Emilie Ringe*[a,b]*

a. Department of Materials Science and Metallurgy, University of Cambridge, 27 Charles Babbage Road, Cambridge CB3 0FS, United Kingdom

b. Department of Earth Sciences, University of Cambridge, Downing Street, Cambridge CB2 3EQ, United Kingdom

METHODS

Materials

Li pellets (99 %), naphthalene (99 %), anthracene (99 %), 1.0 M di-*n*-butyl magnesium ($MgBu_2$) in heptane, poly(vinyl pyrrolidone) (PVP, average molecular weight = 10,000), anhydrous tetrahydrofuran (THF, 99.9 %, containing 250 ppm butylated hydroxytoluene inhibitor), and nitric acid (70 %) were purchased from Sigma-Aldrich and used as supplied. Anhydrous isopropanol (IPA) was purchased from Flurochem, acetone (99.8 %, extra dry) was purchased from Thermo Fisher Scientific, and anhydrous methanol was purchased from VWR Chemicals and used as supplied. All glassware were washed with nitric acid, rinsed with water, dried in a 110 °C oven, and flame-dried under vacuum before use.

Synthesis of Mg NPs

The synthesis of quasi-spherical Mg NPs is a one-pot three-stage process (Figure 1 in the main text). For the first reduction stage, lithium naphthalene dianion (Li_2Napht) was freshly prepared by 45 min sonication (Allendale Ultrasonics Cavitek Professional 100 W, 3 L) of a mixture of Li pellets (28 mg, 4.03 mmol), dry naphthalene (256 mg, 2.00 mmol), PVP (20 mg), and THF (10.75 mL) in a 25 mL Schlenk flask under Ar atmosphere at room temperature. $MgBu_2$ in heptane (1.75 mL, 1.0 M) was then injected quickly into the flask containing Li_2Napht under stirring (550 rpm) at room temperature (**WARNING! $MgBu_2$ is pyrophoric and should be handled under inert conditions**). For the following steps, naphthalene (1282 mg, 10 mmol) and PVP (50 mg) were dissolved in THF (5 mL) by sonicating under Ar atmosphere. The mixture was injected quickly into the reaction flask at 5 min (1 mL) and 65 min (2 mL) after the injection of $MgBu_2$ for the second reduction and etching stages, respectively, unless specified otherwise. For experiments with anthracene in the etching stage, anthracene was used in place of naphthalene for the injection at 65 min.

The reaction was left to stir (550 rpm) under Ar atmosphere until it was quenched after further 1 h by injecting acetone (2 mL). Intermediate products were obtained by quenching the reaction with acetone (2 mL) earlier in the synthesis, or by taking aliquots (0.8 mL) under Ar atmosphere using a glass pipette and quenching in 8 % acetone in THF (4 mL). The resulting solid product was recovered by centrifugation (8,000 rcf, 10 min) and residual byproducts were removed through cycles of centrifugation (10,000 rcf, 10 min) and redispersion steps in THF twice, acetone, IPA, anhydrous methanol, and IPA in the listed order, before the product was finally redispersed in the original volume of IPA.

Characterisation of Mg NPs

Scanning electron microscopy (SEM) of Mg nanoparticles (NPs) drop-cast on Si wafers was performed on ZEISS GeminiSEM operated at 5.00 kV and using an in-lens detector for secondary electron imaging. For size measurements, NPs were separated from the background by a machine learning algorithm on ilastik 1.4.[1] SEM images were acquired in groups of equal magnification and combined into the hdf5 format using Fiji. Using first the Pixel Classification workflow, the algorithm was trained for particle and background using a brush tool on more than 10 randomly selected SEM images containing 5 to 10 Mg NPs each. The resulting pixel prediction maps were fed into the Object Classification workflow, where the software was trained to identify quasi-spheres. The Pixel and Object Classifications were then performed consecutively on a group of SEM images. The hysteresis method was used for threshold and size filtration, for which the values were adjusted depending on the contrast and magnification of the batch of images. The object prediction maps were then loaded into Fiji, where the scale of the images was set, maps converted to binary, and NPs fitted to ellipses using Analyze Particles. The lengths of the major and minor axes from each ellipse were exported as a table, with the former used when reporting sizes.

Ultraviolet-visible-near infrared (UV-Vis-NIR) spectroscopy was performed on Thermo Fisher Evolution 220 UV-visible spectrophotometer with Mg NPs suspended in IPA at room temperature, in polymethyl methacrylate (PMMA) semimicro cuvettes.

Dynamic light scattering (DLS) was performed using a Malvern Zetasizer Nano ZSP equipped with a 633 nm laser. Samples of Mg NPs suspended in IPA were prepared in PMMA micro cuvettes and measured at 20 °C following a 30 sec equilibration time. The refractive index and absorption of Mg was set to 0.351 and 0.250, respectively. The viscosity and refractive index of IPA was set to 2.3702 cP and 1.378, respectively. Three measurements were averaged for each sample.

Scanning transmission electron microscopy high-angle annular dark field (HAADF-STEM) images were collected on a FEI Tecnai Osiris operated at 200 kV and equipped with a Gatan UltraScan1000XP (2048 by 2048 pixel) camera. Samples were prepared by drop-casting a dilute colloid of Mg NPs onto a carbon film supported by a copper mesh grid.

Powder X-ray diffraction (XRD) was performed using a Bruker D8 DAVINCI diffractometer equipped with a Cu Kα source and a position sensitive detector (LynxEye EX) in coupled theta/2theta mode, and using 0.01° step size. Samples were prepared by drop-casting a concentrated colloid of Mg NPs on to the centre of a Bruker Si low background sample holder. The background was subtracted using an automatic fitting and subtraction function in the DIFFRAC.EVA V7.2 software.

Inductively coupled plasma optical emission spectroscopy (ICP-OES) analysis was performed on Thermo Fisher Scientific iCAP 7400 Duo ICP-OES Analyzer. Prior to the analysis, Mg NPs were digested in an aqueous matrix using dilute nitric acid then diluted to ~1 ppm (mg L^{-1}) with deionised water.

Numerical Methods

Extinction cross sections for spheres of varying size and MgO shell thicknesses were obtained by numerical calculations through solving Maxwell's equations via a transfer-matrix method using STRATIFY.[2] Extinction cross sections for a faceted NP and a sphere of equal volume, with and without a 9 nm MgO, were obtained by numerical calculations through solving Maxwell's equations in the discrete dipole approximation using DDSCAT.[3] The interdipole distance was set to 3 nm and an orthogonally polarised light was used as the incident field. For all simulations, the frequency dependent refractive index of Mg was taken from Pehlivan et al.[4] and of MgO, from Palik[5] while the refractive index of the surrounding medium (IPA) was set to 1.3772.

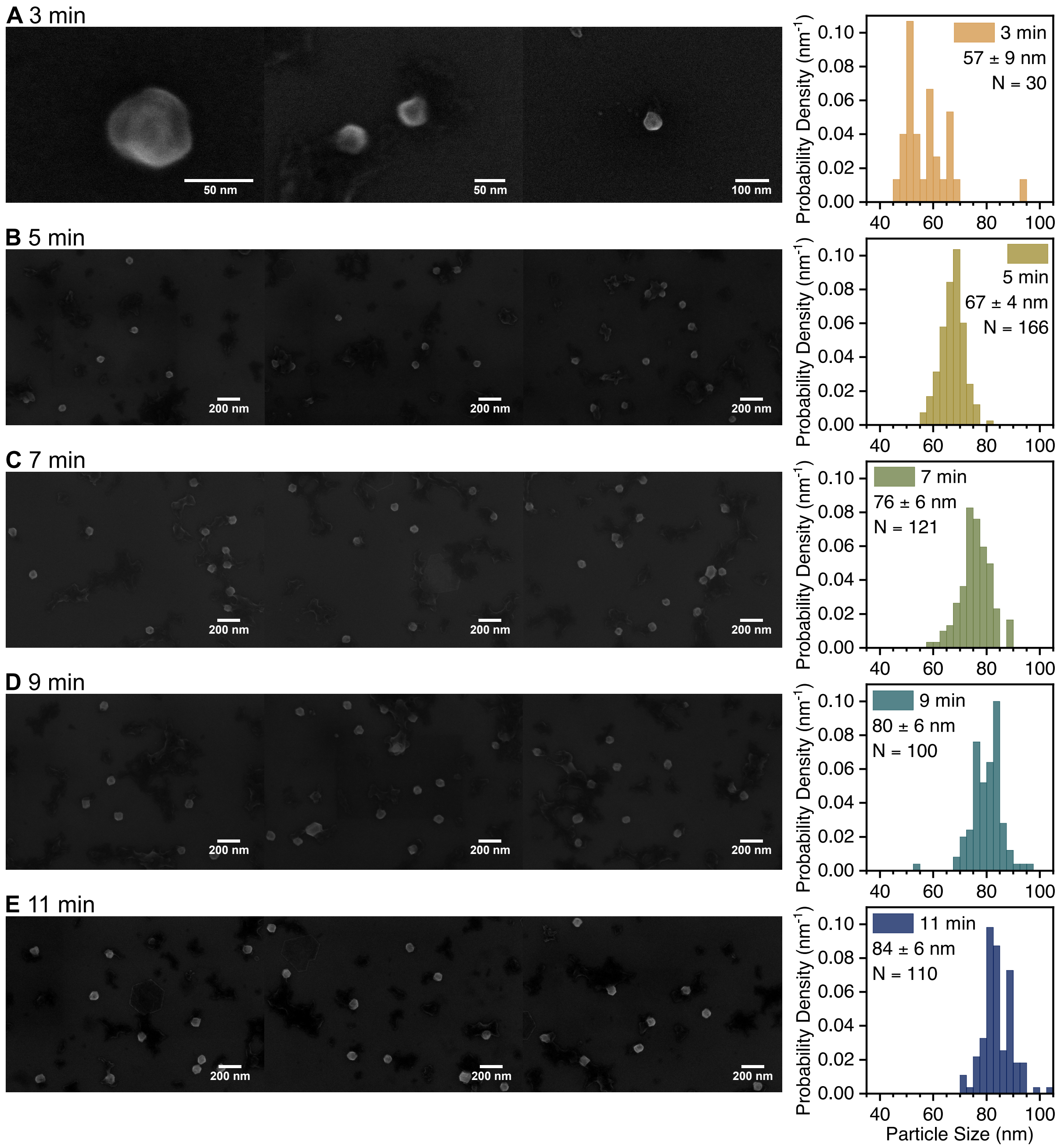

Figure S1. Mg NPs grow during the first reduction stage. (A – E) Representative SEM images of Mg NPs sampled at labelled reaction times throughout the first reduction stage and their size distribution histograms showing the diameter.

Calculation of the average volume and number of Mg NPs from reaction aliquots in the synthesis of quasi-spherical Mg NPs

Assuming a spheroidal geometry with two short, equal minor axes and one long (major) axis, the volume of each NP measured from SEM was calculated using:

$$V = \frac{4}{3}\pi a b^2 \quad (1)$$

where a is the length of the major axis and b is the length of the minor axis. The volumes were then averaged across all measured particles in the aliquot (Table S1). The average mass of NPs was then calculated using the average volume and the density of Mg, 1.738 g cm^{-3}.[6] Finally, the number of NPs was calculated by dividing the Mg concentration from ICP-OES by the average NP mass (Table S1).

Table S1. Average NP volumes calculated using size measurements from SEM, concentration of Mg from ICP-OES, and calculated number of Mg NPs in each aliquots collected across the three stages of the quasi-spherical Mg NP synthesis.

Stage	Reaction Time (min)	Average NP Volume (nm^3)	Mg Concentration (mg/L)	Number of NPs (mL^{-1})
First Reduction	3	6.62×10^5	164	1.42×10^{11}
	5	1.01×10^6	205	1.17×10^{11}
	7	1.44×10^6	208	8.32×10^{10}
	9	1.61×10^6	264	9.41×10^{10}
	11	1.93×10^6	263	7.83×10^{10}
	1	1.69×10^6	377	1.28×10^{11}

Second Reduction (5 min in the first reduction stage)	5	9.36×10^6	682	4.19×10^{10}
	10	1.28×10^7	892	4.00×10^{10}
	15	1.37×10^7	940	3.94×10^{10}
	20	1.43×10^7	1060	4.25×10^{10}
	25	1.38×10^7	1080	4.52×10^{10}
Etching (5 and 60 min in the first and second reduction stages, respectively)	1	1.36×10^7	814	3.44×10^{10}
	30	1.20×10^7	730	3.49×10^{10}
	60	1.15×10^7	703	3.53×10^{10}

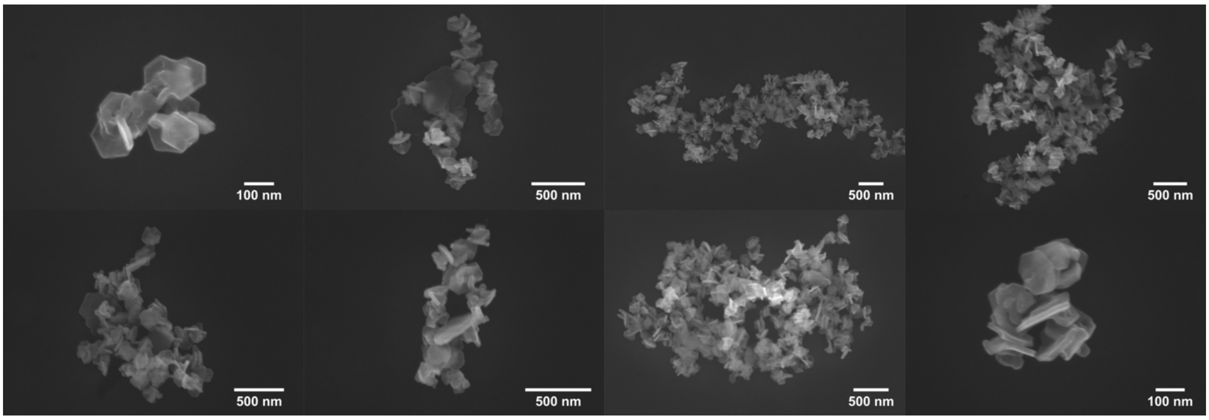

Figure S2. Growing Mg NPs in the first reduction stage beyond 11 min results in a mixture of faceted NPs and hexagonal platelets. SEM images of Mg NPs obtained from a 60 min reaction in the first reduction stage.

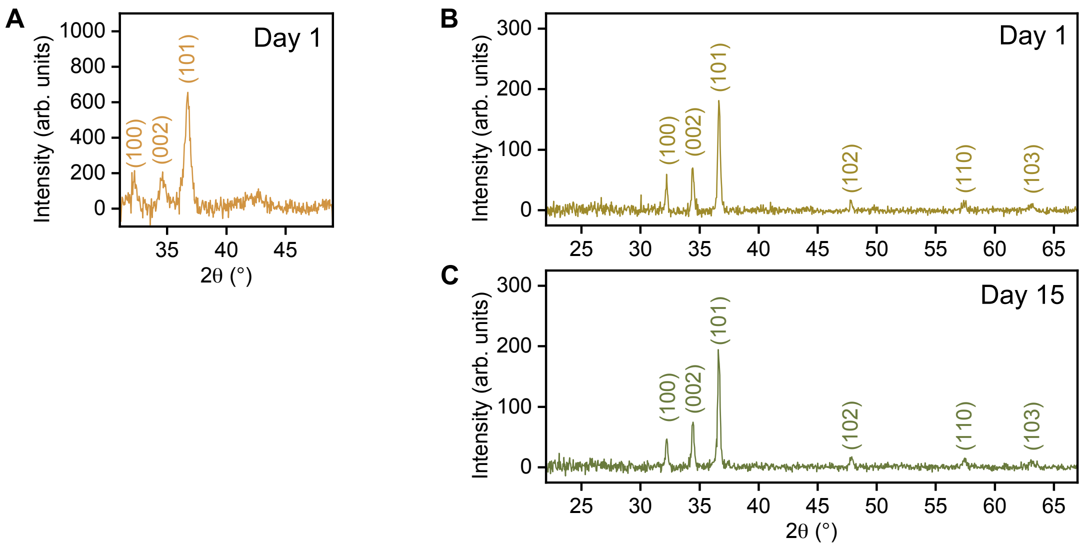

Figure S3. Synthesised Mg NPs are stable against oxidation in air. XRD patterns of faceted Mg NPs of (A) 67 and (B) 167 nm in diameter. (C) XRD pattern of 167 nm faceted Mg NPs after they were left dried in ambient air for 15 days. All patterns show peaks corresponding to HCP Mg (PDF 00-035-0821).

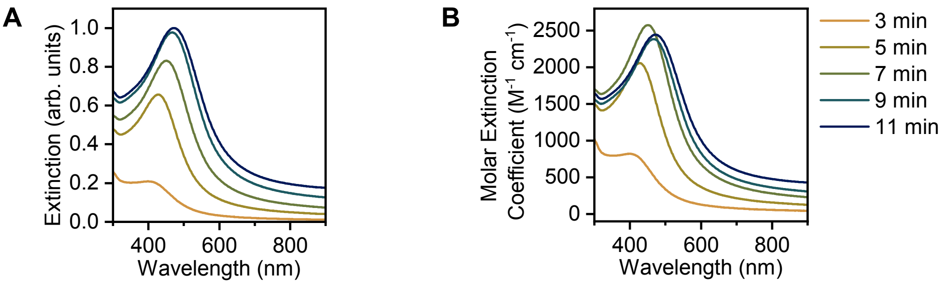

Figure S4. Non-normalised UV-Vis-NIR extinction spectra of Mg NPs throughout the first reduction stage. (A) Spectra of as-synthesised Mg NPs with equal dilutions and (B) those divided by the molarity of Mg in each colloid.

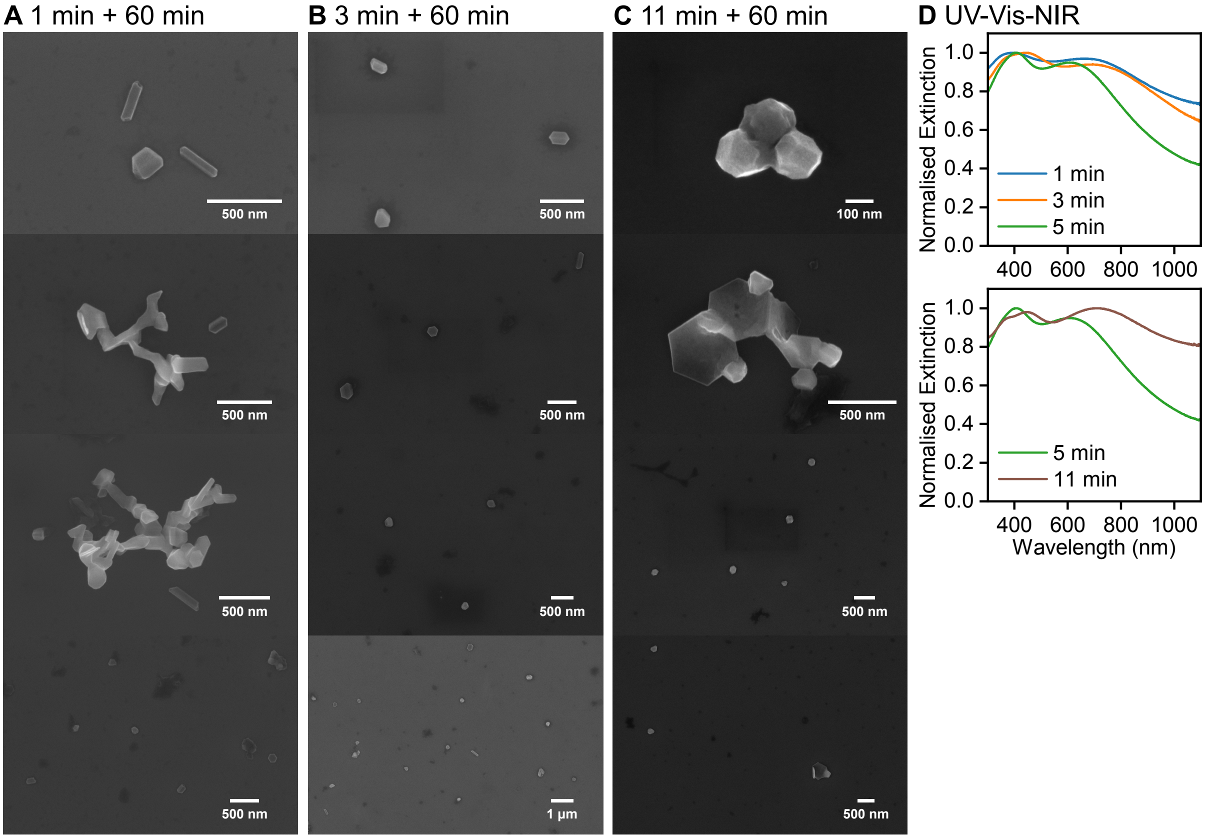

Figure S5. Syntheses with a first reduction stage shorter than 5 min or longer than 9 min result in a heterogenous colloid of Mg NPs. SEM images of faceted Mg NPs grown by (A) 1, (B) 3, and (C) 11 min reactions in the first reduction stage, followed by 60 min of reaction in the second reduction stage, and (D) UV-Vis-NIR spectra of the resulting NP colloids, with that of the 5 min reaction shown as a reference.

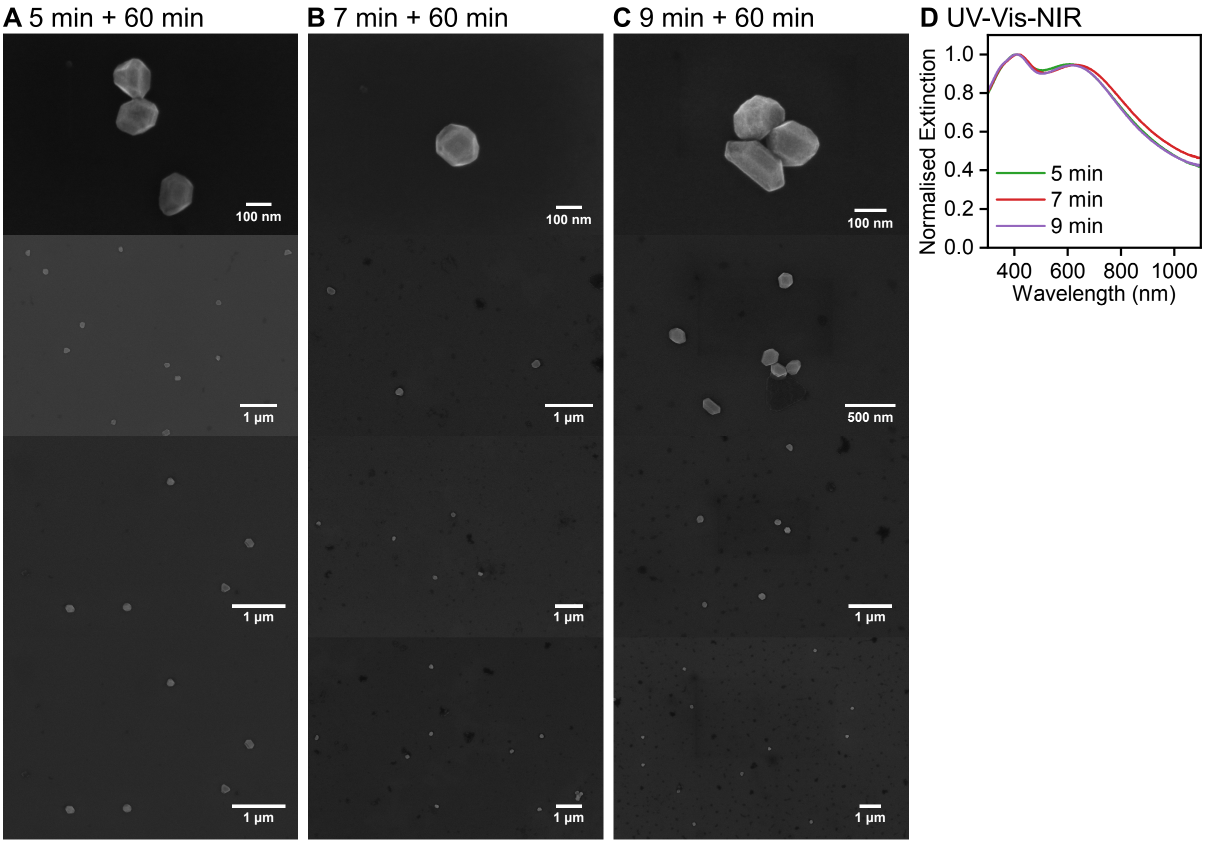

Figure S6. Reaction times between 5 and 9 min in the first reduction stage result in a homogenous colloid of Mg NPs. SEM images of faceted Mg NPs grown by (A) 5, (B) 7, and (C) 9 min reactions in the first reduction stage, followed by 60 min of reaction in the second reduction stage and (D) UV-Vis-NIR spectra of the resulting NP colloids.

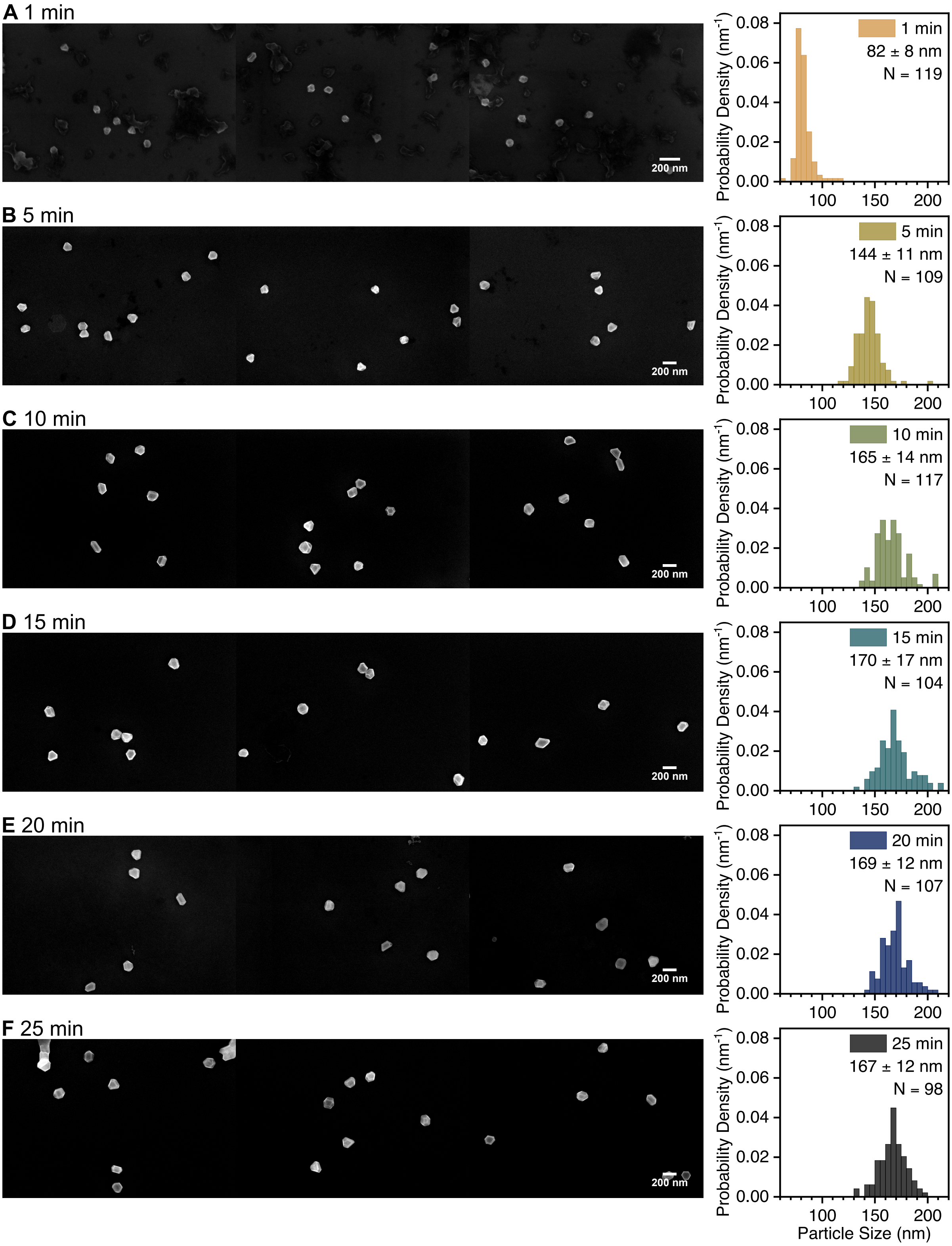

Figure S7. Mg NPs grow during the second reduction stage. (A – F) Representative SEM images of Mg NPs sampled at labelled reaction times throughout the second reduction stage, following a 5 min reaction in the first reduction stage, and their size distribution histograms showing the diameter.

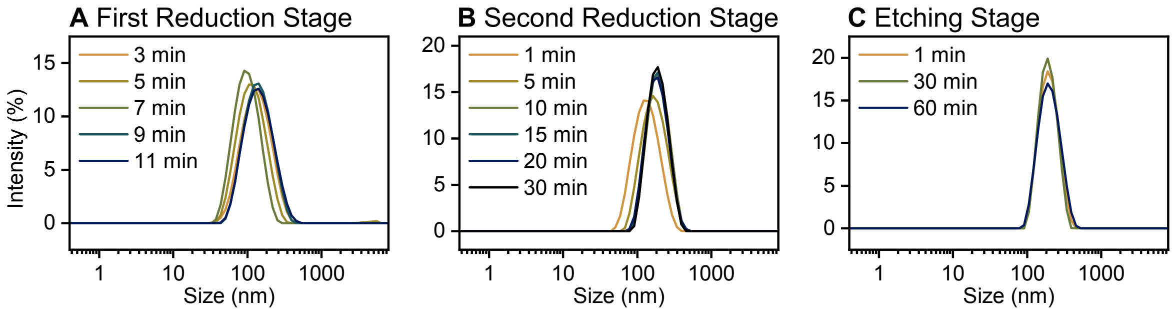

Figure S8. Change in size of Mg NPs measured by DLS. Size distribution of Mg NPs obtained from DLS for aliquots collected during the (A) first reduction, (B) second reduction – 5 min first stage, and (C) etching stage – 5 and 60 min in first and second stages, respectively, in the synthesis of quasi-spherical Mg NPs.

Table S2. Average sizes of Mg NPs obtained by DLS for aliquots collected during the first reduction stage, second reduction stage, and etching stage in the synthesis of quasi-spherical Mg NPs. Sizes are reported as intensity weighted mean hydrodynamic diameters (Z-Average).

Stage	Reaction Time (min)	Z-Average (nm)
First Reduction	3	113
	5	101
	7	88
	9	127
	11	128
Second Reduction (5 min in the first reduction stage)	1	122
	5	159
	10	183
	15	176

	20	175
	25	180
Etching (5 and 60 min in the first and second reduction stages, respectively)	1	188
	30	183
	60	189

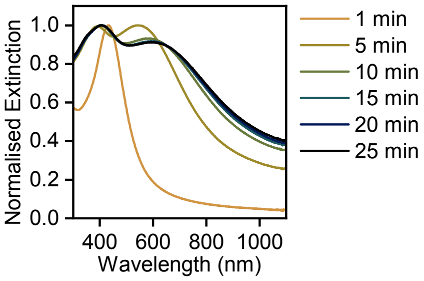

Figure S9. UV-Vis-NIR extinction spectra from all aliquots collected during the second reduction stage in the growth of faceted Mg NPs, following a 5 min reaction in the first reduction stage.

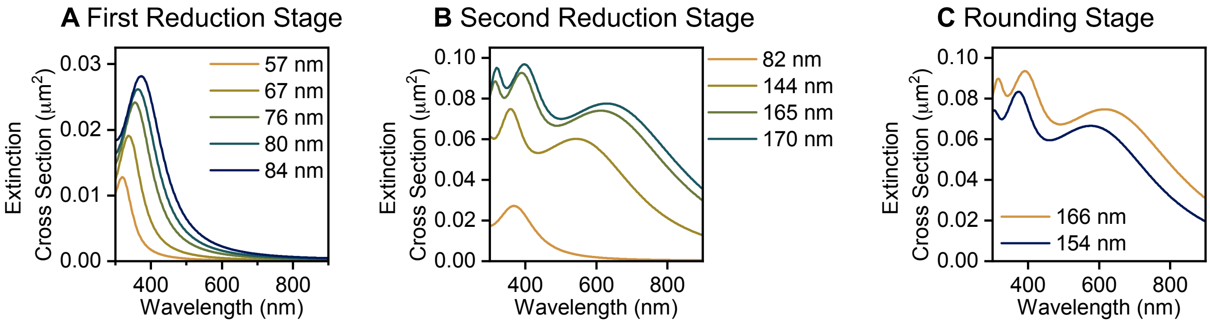

Figure S10. Simulated extinction cross sections of spherical Mg NPs using Mie theory. Sizes follow those observed during the (A) first reduction, (B) second reduction, and (C) etching stages in the synthesis of quasi-spherical Mg NPs; all include a 10 nm MgO layer surrounding a Mg^0 core.

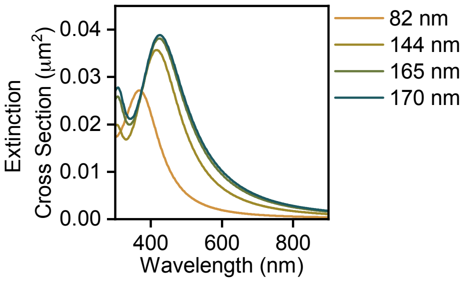

Figure S11. Simulated extinction cross sections of spherical Mg NPs of increasing oxide layer thickness using Mie theory. The diameter of the Mg^0 core was fixed at 62 nm for all sizes and the core was surrounded by an MgO layer accounting for the remaining diameter.

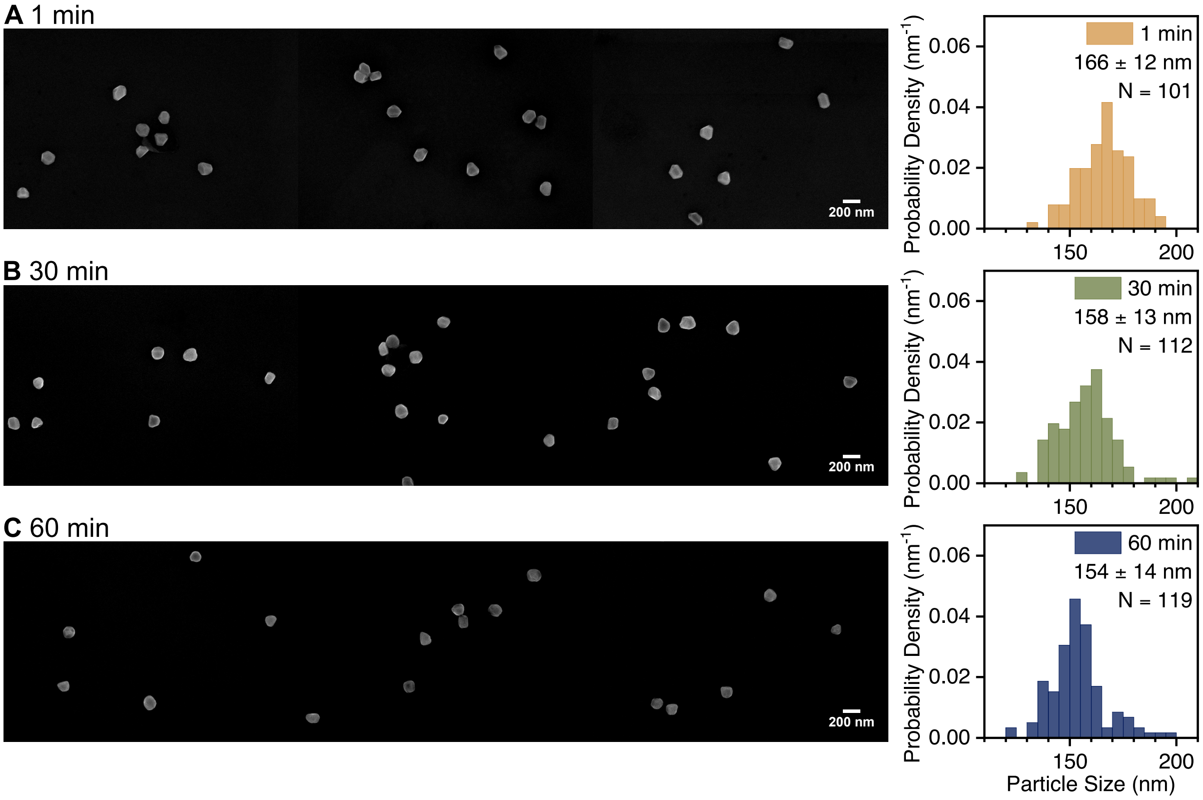

Figure S12. The size of Mg NPs decreases during the etching stage. (A – C) Representative SEM images of Mg NPs sampled at labelled reaction times throughout the etching reduction stage, following 5 and 60 min reaction times in first and second reduction stages, respectively, and their size distribution histograms showing the diameter.

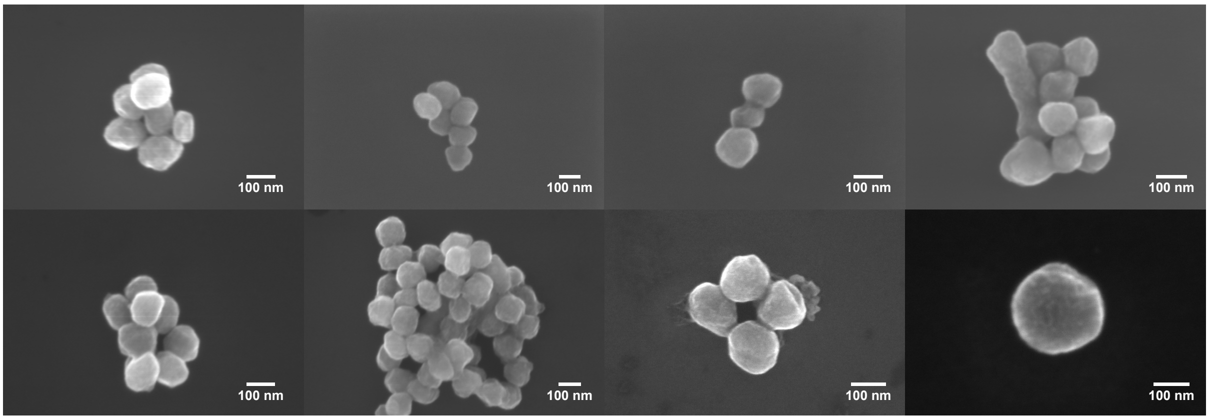

Figure S13. Quasi-spherical Mg NPs are also obtained from four other faceted Mg NP batches. SEM images of Mg NPs obtained at the end of the etching stage.

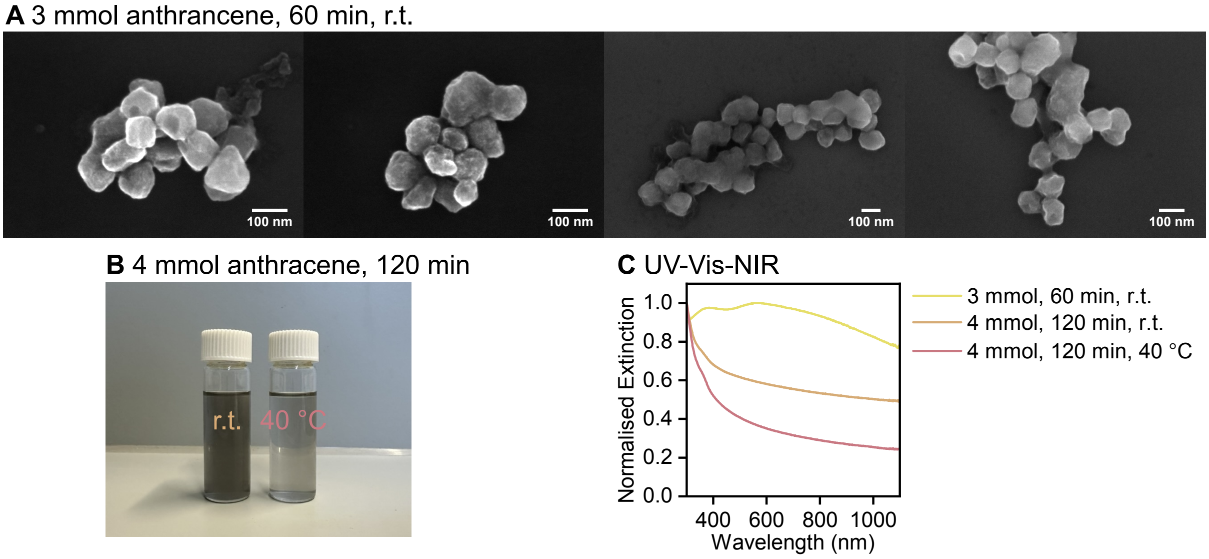

Figure S14. Anthracene significantly etches Mg NPs. (A) SEM images of colloidal Mg NPs etched with 3 mmol of anthracene for 60 min at room temperature, (B) photograph of colloidal Mg NPs etched with 4 mmol of anthracene for 120 min at room temperature and at 40 °C, and (C) their UV-Vis-NIR extinction spectra.

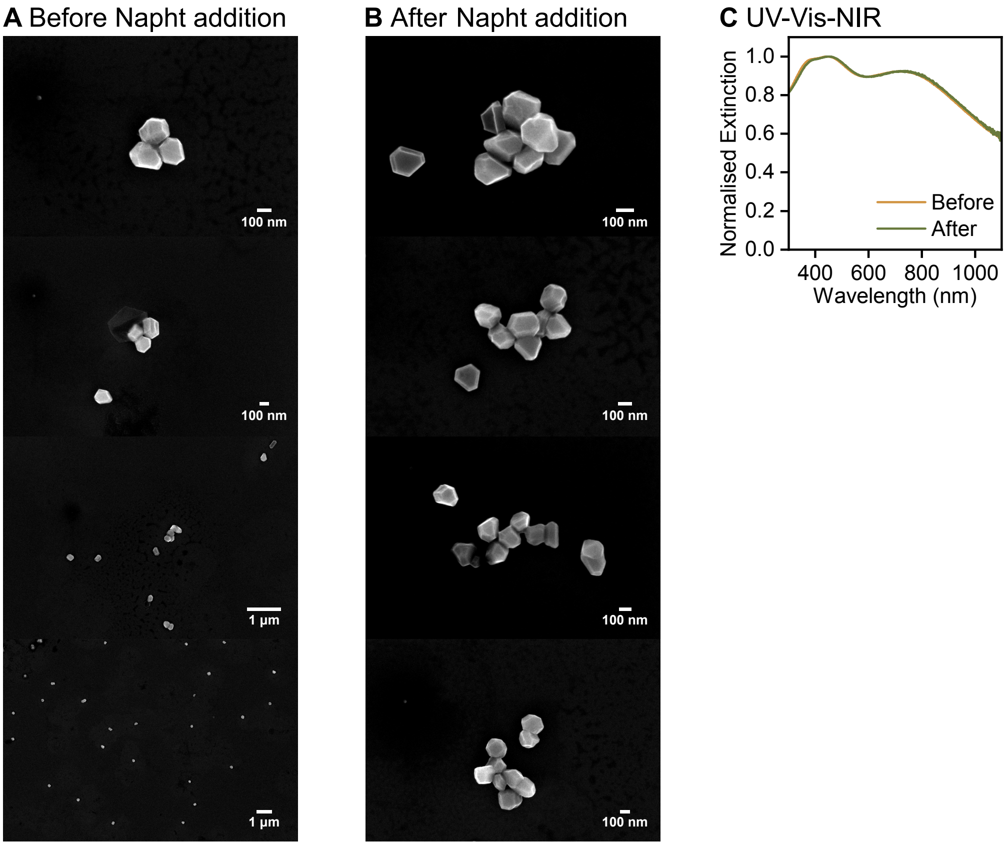

Figure S15. Addition of naphthalene to Mg NPs after oxide formation (through quenching and cleaning) does not etch the NPs. SEM images of Mg NPs (A) before and (B) after the addition of naphthalene, and (C) their UV-Vis-NIR spectra.

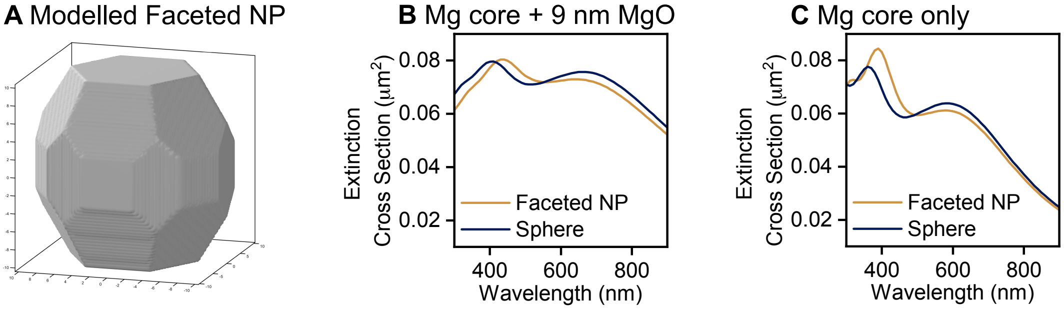

Figure S16. The dipolar LSPR does not shift upon shape change alone. (A) Modelled faceted NP shape of 166 nm in size used for simulation. Simulated extinction cross section of a faceted NP (as in A) and of a spherical NP with equal volume in the (B) presence and (C) absence of a 9 nm surface MgO layer.

References

1. S. Berg, D. Kutra, T. Kroeger, C. N. Straehle, B. X. Kausler, C. Haubold, M. Schiegg, J. Ales, T. Beier, M. Rudy, K. Eren, J. I. Cervantes, B. Xu, F. Beuttenmueller, A. Wolny, C. Zhang, U. Koethe, F. A. Hamprecht and A. Kreshuk, *Nat. Methods*, 2019, **16**, 1226–1232.
2. I. L. Rasskazov, P. S. Carney and A. Moroz, *OSA Continuum*, 2020, **3**, 2290–2306.
3. B. T. Draine and P. J. Flatau, *J. Opt. Soc. Am. A*, 1994, **11**, 1491–1499.
4. Z. S. Pehlivan, A. Ten, T. M. R. Wayman and E. Ringe, *APL Photonics*, 2024, **9**, 071302.
5. E. D. Palik, *Handbook of Optical Constants of Solids*, Academic Press, 1998, vol. 3.
6. W. Jahnen-Dechent and M. Ketteler, *Clin. Kidney J.*, 2012, **5**, i3–i14.
